# Metabolic Adaptation to Chronic Inhibition of Mitochondrial Protein Synthesis in Acute Myeloid Leukemia Cells

**DOI:** 10.1371/journal.pone.0058367

**Published:** 2013-03-08

**Authors:** Bozhena Jhas, Shrivani Sriskanthadevan, Marko Skrtic, Mahadeo A. Sukhai, Veronique Voisin, Yulia Jitkova, Marcela Gronda, Rose Hurren, Rob C. Laister, Gary D. Bader, Mark D. Minden, Aaron D. Schimmer

**Affiliations:** 1 The Princess Margaret Hospital and The Ontario Cancer Institute, University Health Network, Toronto, Canada; 2 The Donnelly Centre, University of Toronto, Toronto, Canada; 3 Department of Molecular Genetics, University of Toronto, Toronto, Canada; University of Pecs Medical School, Hungary

## Abstract

Recently, we demonstrated that the anti-bacterial agent tigecycline preferentially induces death in leukemia cells through the inhibition of mitochondrial protein synthesis. Here, we sought to understand mechanisms of resistance to tigecycline by establishing a leukemia cell line resistant to the drug. TEX leukemia cells were treated with increasing concentrations of tigecycline over 4 months and a population of cells resistant to tigecycline (RTEX+TIG) was selected. Compared to wild type cells, RTEX+TIG cells had undetectable levels of mitochondrially translated proteins Cox-1 and Cox-2, reduced oxygen consumption and increased rates of glycolysis. Moreover, RTEX+TIG cells were more sensitive to inhibitors of glycolysis and more resistant to hypoxia. By electron microscopy, RTEX+TIG cells had abnormally swollen mitochondria with irregular cristae structures. RNA sequencing demonstrated a significant over-representation of genes with binding sites for the HIF1α:HIF1β transcription factor complex in their promoters. Upregulation of HIF1α mRNA and protein in RTEX+TIG cells was confirmed by Q-RTPCR and immunoblotting. Strikingly, upon removal of tigecycline from RTEX+TIG cells, the cells re-established aerobic metabolism. Levels of Cox-1 and Cox-2, oxygen consumption, glycolysis, mitochondrial mass and mitochondrial membrane potential returned to wild type levels, but HIF1α remained elevated. However, upon re-treatment with tigecycline for 72 hours, the glycolytic phenotype was re-established. Thus, we have generated cells with a reversible metabolic phenotype by chronic treatment with an inhibitor of mitochondrial protein synthesis. These cells will provide insight into cellular adaptations used to cope with metabolic stress.

## Introduction

Eukaryotic cells have two separate genomes; nuclear DNA organized in chromosomes, and the 16.6 kb circular mitochondrial DNA located within the mitochondria. The mitochondrial genome encodes two rRNAs, 22 t-RNAs and 13 of the 90 proteins in the mitochondrial respiratory chain [1]. Translation of the mitochondrially-encoded proteins occurs in the mitochondrial matrix, and involves distinct protein synthesis machinery, including unique mitochondrial ribosomes, initiation and elongation factors and t-RNAs. Thus, mitochondria regulate oxidative phosphorylation through both transcription and translation.

Depletion of mitochondrial DNA produces rho-zero cells that have no mitochondrially translated proteins. As such, these cells lack a functional respiratory chain and cannot derive energy from oxidative phosphorylation. Instead, these cells rely on glycolysis for their energy supply. Traditionally, generating rho-zero cells requires a prolonged exposure of a parental cell line to cationic lipophilic agents such as ethidium bromide [2] or chemotherapeutic agents such as ditercalinium [3] to inhibit mitochondrial DNA replication and, over time, permanently deplete mitochondrial DNA. Prolonged exposure to ethidium bromide or chemotherapeutic agents, however, can also damage nuclear DNA, thus potentially confounding the experimental results. In addition, rho-zero cells generated through these approaches have irreversible mitochondrial DNA depletion and irreversible changes in their metabolism.

Recently, we reported that the anti-bacterial agent tigecycline preferentially induces death in acute myeloid leukemia (AML) cells and AML stem cells through a mechanism related to inhibition of mitochondrial protein synthesis [4]. Impairment of mitochondrial protein synthesis led to the dysfunction of electron transport chain and inhibition of the oxidative phosphorylation pathway. We also demonstrated that the heightened sensitivity of AML cells to inhibition of mitochondrial translation was derivative of increased mitochondrial mass and greater dependence on oxidative phosphorylation in these cells compared to normal hematopoietic cells. To better understand mechanisms of sensitivity and resistance to inhibitors of mitochondrial protein synthesis, we treated TEX leukemia cells [5] with increasing concentrations of the mitochondrial protein synthesis inhibitor tigecycline and over time selected a population of resistant cells. Tigecycline resistant TEX cells had repressed mitochondrial translation and undetectable levels of oxidative phosphorylation, but maintained their mitochondrial DNA. These cells were dependent on glycolysis for their energy supply and molecularly they upregulated HIF1α. Strikingly, the metabolic phenotype was reversible, as withdrawal of tigecycline restored mitochondrial protein synthesis and oxidative phosphorylation. Thus, by generating cells resistant to mitochondrial protein synthesis, we have gained further insights into how cells cope with metabolic stress.

## Materials and Methods

### Cell Culture

TEX human leukemia cells (a gift from Dr. J. Dick, Toronto, Canada) were derived from lineage depleted human cord-blood cells retrovirally transduced with TLS_ERG oncogene [5]. RTEX+TIG cells are a subclone of TEX selected by *in vitro* treatment with tigecycline [5]. TEX and RTEX+TIG were maintained in IMDM, 15% FBS, 1% penicillin-streptomycin, 20 ng/mL SCF, 2 ng/mL IL-3 and 2 mM L-glutamine. Cells were incubated at 37°C in a humidified air atmosphere supplemented with 5% CO_2_. For hypoxia experiments, cells were transferred to hypoxic culture chambers (MACS VA500 microaerophilic workstation, H35 HypoxyWorkStation; Don Whitley Scientific, Fredrick, MD, USA). The atmosphere inside the chambers consisted of 5% H_2_ 5% CO_2_, 0% or 0.2% O_2_ and residual N_2_.

### Selection of Tigecycline-resistant Cells

Tigecycline-resistant cells (RTEX+TIG) were selected by a step-wise exposure to increasing concentrations of tigecycline. Wild type TEX cells were initially exposed to 6 µM tigecycline and the dose gradually increased to 24 µM over a 4 month period. A population of resistant cells were selected and maintained in medium supplemented with 24 µM tigecycline.

### Cell Growth and Viability Assay

Cell death was measured by Annexin V-fluorescein isothiocyanate (FITC) and Propidium Iodide (PI) (Biovision Research Products, Mountain View, CA) staining using flow cytometry according to the manufacturer’s instructions. Cell growth and viability was measured with the Sulforhodamine B assay as previously described [6], [7] and by trypan blue staining.

### Determination of Intracellular Concentrations of Tigecycline

Intracellular tigecycline was measured in TEX and RTEX + TIG cells by HPLC with UV detection (350 nm). Cellular proteins were precipitated by addition of 20 µL 100%-trichloroacetic acid containing 200 µg/mL minocycline as an internal standard. Then the aqueous phase was loaded on a Symmetry C18 column (3.9*150 mm, 5 µm). Tigecycline and minocycline were separated by 25∶75 (v/v) acetonitrile-phosphate buffer (0.023 M, pH 3.0) containing 4 mM 1-octanesulfonic acid.

### Immunoblotting

Total cell lysates were prepared from cells as described previously [8]. Briefly, cells were washed twice with phosphate buffered saline pH 7.4 and suspended in lysis buffer (1.5% n-dodecyl β-maltoside (Sigma Aldrich, St. Louis, MO)) containing protease inhibitor tablets (Complete tablets; Roche, IN). Protein concentrations were measured by the DC Protein assay (Bio Rad, Hercules, CA). Equal amounts of protein were subjected to sodium dodecyl sulphate (SDS)-polyacrylamide gels followed by transfer to nitrocellulose membranes. Membranes were probed with anti-Cox-1 1∶1000 (Santa Cruz Biotechnology Inc), anti-Cox-2 1∶500 (Santa Cruz Biotechnology Inc), anti-Cox-4 1∶2000 (Santa Cruz Biotechnology Inc), anti-HIF1α 1∶1000 (Cell signaling Technology), anti-α-tubulin 1∶2000 (Sigma Aldrich, St. Louis, MO), anti-β-actin 1∶1000 (Cell signaling Technology) and secondary antibodies from GE Health (IgG peroxidase linked species-specific whole antibody). Detection was performed by the enhanced chemical luminescence method (Pierce, Rockford, IL).

### Enzymatic Activity of the Respiratory Chain Complexes

Complex activity was analyzed from mitochondria–enriched pellet, prepared by cell lysis and centrifugation, followed by freezing at –80°C for 7 days. The frozen pellet was then re-suspended in Dodecyl–D–maltoside, analyzed for protein concentration and citrate synthase activity. Complex II activity was measured by monitoring malonate-sensitive reduction of 2,6-dichloroindophenol when coupled to complex II-catalyzed reduction of decylubiquinol at 600 nm, with 750 nm as the reference wavelength [9]. Complex III activity was assessed with a modified method described by Birch-Machin et al [10] and Krahenbul et al [11]. Complex III-specific activity was measured by following the increase in absorbance due to the reduction of cytochrome c at 550 nm, with 580 nm as the reference wavelength. Complex IV activity was measured by KCN-sensitive oxidation of ferrocytochrome c at 550 nm, with 540 nm as the reference wavelength [12]. Ferrocytochrome c was prepared by reducing cytochrome c with sodium ascorbate followed by dialysis for 24 hours [13]. Citrate synthase activity was measured based on the chemical coupling of CoASH, released from acetyl-CoA during the enzymatic synthesis of citrate to DTNB (Ellman’s reagent, 5,5′-dithiobis(2-nitrobenzoic acid), and the release of the absorbing mercaptide ion was monitored at 412 nm [14]. The enzyme activity of Complexes II, III, and IV was normalized to citrate synthase activity.

### Oxygen Consumption and Extracellular Acidification Rates

The rates of oxygen consumption and extracellular acidification were measured using a Seahorse XF96 analyzer as per manufacturer’s instructions (Seahorse Bioscience, North Billerica, MA, USA). Cells were washed, re-suspended with un-buffered medium, and seeded at 100,000 cells/well in XF96 plates. Cells were then equilibrated to the un-buffered medium for 45 min at 37°C in a CO_2_-free incubator and transferred to the XF96 analyzer. The rates of oxygen consumption (OCR) and extracellular acidification (ECAR) were measured simultaneously for 3 minutes.

### Lactate Production

Metabolites were extracted from 2×10^7^ cells with 80% MeOH and the solvent was removed in a centrifugal vacuum concentrator at room temperature. The resulting powder was re-dissolved in 120 µL of NMR buffer (50 mM Na_2_HPO_4_, pH 7.0, 0.1%NaN_3_), DSS was added to a final concentration of 0.5 mM and the sample was transferred to a 3 mm NMR tube. One dimensional ^1^HNMR spectra were acquired at 298K on a Bruker Avance 800 MHz spectrometer equipped with a triple resonance cryoprobe. A total of 128 scans were acquired over a 14 ppm spectral width using a 90° pulse and a 2 s recycle delay. The data were processed and the metabolites were quantified using the Chenomx NMR suite 7.1 (Edmonton, AB).

### ATP Quantification

The quantity of ATP present in cells was determined using the CellTitre-Glo Luminescent Cell Viability Assay (Promega, Madison, WI), according to the manufacture’s instructions.

### Mitochondrial Mass Measurements

To assess mitochondrial DNA (mtDNA) copy number, genomic DNA was extracted from RTEX+TIG and TEX cells using the DNAeasy Blood and Tissue kit (Qiagen MD, USA). The relative mtDNA copy number was determined by a real-time polymerase chain reaction (qPCR), and compared relative to nuclear DNA as previously described [15]. The primer sequences were forward primer (ND1-F), 5′-CCCTAAAACCCGCCACATCT-3′; reverse primer (ND1-R), 5′-GAGCGATGGTGAGAGCTAAGGT-3′, forward primer (HGB-F), 5′-GTGCACCTGACTCCTGAGGAGA-3′; reverse primer (HGB-R), 5′-CCTTGATACCAACCTGCCCAG-3′.

To determine mitochondrial mass, cells were stained with 50 nM of Mitotracker Green FM (Invitrogen, Carlsbad, CA) in PBS buffer at 37°C for 30 minutes, and then re-suspended in PBS. Samples were analyzed on a BD FACSCalibur flow cytometer. The median fluorescence intensity in the FL1 channel was divided by the Forward Scatter (FSC) measurement as an estimate of mitochondrial mass. Data were analyzed with FlowJo version 7.7.1 (TreeStar).

### Determination of Mitochondrial Membrane Potential

To measure mitochondrial membrane potential, cells were washed twice with PBS and incubated with 2 µM of 5,5′,6,6′-tetrachloro-1,1′,3,3′-tetraethyl benzimidazolylcarbocyanine iodide (JC-1, Invitrogen) for 30 minutes at 37°C. Each sample was then washed with 1 mL PBS and re-suspended in 500 µL PBS prior to analysis on a BD FACSCalibur flow cytometer. Samples were excited at 488 nm and emission was collected at 526 nm (FL1) and 595 nm (FL2). Analysis was conducted using FlowJo version 7.7.1 (TreeStar, Ashland, OR). Mitochondrial membrane potential (FL2/FL1) was calculated as the emission from the red channel divided by emission from the green channel.

### Transmission Electron Microscopy

The mitochondrial morphology of TEX, RTEX+TIG and RTEX-TIG cells was assessed by transmission electron microscopy (TEM). Cells were harvested, and fixed with a Graham-Karnovsky’s technique as previously described [16] in 4% paraformaldehyde plus 1% glutaraldehyde in 0.1 M phosphate buffer pH 7.2 (PB) for 1 hour at room temperature. Cells were washed with PB 3 times for 30 minutes. Next, cells were post-fixed with 1% osmium tetroxide buffered with PB for 1 hour and washed again using distilled water twice for 30 minutes, dehydrated with ethanol, washed with propylene oxide, and treated with epoxy resin, which was polymerized at 60°C for 48 hours. Solid epoxy blocks were sectioned on a Reichert Ultracut E microtome to 90 nm thickness, collected on 300 mesh copper grids and counterstained with uranyl acetate and lead citrate. A Hitachi H7000 (Hitachi, Tokyo, Japan) transmission electron microscope was used to evaluate the sections at an accelerating voltage of 75 kV.

### RNA Sequencing

Total RNA (10 µg) was harvested from TEX and RTEX+TIG cell lines and was sequenced (total reads: TEX, 53,844,949; RTEX+TIG, 53,345,549; matched reads: TEX, 48,086,490; RTEX+TIG, 47,385,961) to yield 50 base pair paired-end reads, using the Illumina GA2X sequencing platform (Illumina), by the Clinical Genomics Centre (the University Health Network/Mt. Sinai Hospital Gene Profiling Facility, Toronto). Data were processed utilizing the human reference genome, v37.2, in the NextGENe analysis environment (v2.14; SoftGenetics) to yield reads per kilobase of exon model per million mapped reads (RPKM) for each identified transcript, and normalized to determine a fractional abundance score for each transcript in each sample. Total RPKM values were comparable between samples (TEX: 93,657.32; RTEX+TIG: 90,663.42). Fold-differences were computed; RPKM values were log2 transformed and the ratio between the RTEX and TEX was calculated as follows: log (FC) = log (RPKM_RTEX+TIG) –log (RPKM_TEX).

Gene expression data were analyzed using gene set enrichment analysis GSEA^1^ [17] with parameters set to 2000 gene-set permutations and gene-sets size between 8 and 500. Genes were ordered using the logFC comparing the RTEX+TIG versus the TEX cells. The gene-sets included in the GSEA analyses were obtained from KEGG, MsigDB-c2, NCI, BioCarta, IOB, Netpath, HumanCyc, Reactome and the Gene Ontology (GO) databases, updated May 2012 (http://baderlab.org/GeneSets). An enrichment map (version 1.2 of Enrichment Map software [18]) was generated for each comparison using enriched gene-sets with a nominal p-value <0.05 and the overlap coefficient set to 0.5.

An unbiased analysis of promoter sequences for differentially expressed genes was undertaken using the oPOSSUM v3.0 (http://opossum.cisreg.ca/oPOSSUM3/; [19]) software tool. This tool maps known transcription factor binding motifs onto promoter sequences of co-expressed genes, in order to determine whether a given transcription factor binding site is over-represented within a dataset. 2000 bp upstream sequences of up-regulated genes were analyzed using oPOSSUM 3.0 Sequence-based Single Site Analysis (default parameters). A custom background sequence set that fits %GC content of the analyzed sequences was built using open chromatin regions from the ENCODE mappable genome project (CRG 36mer).

### Measurement of Regulators of Mitochondrial Mass

The levels of NRF1, TUFM, TFAM, POLG, HIF1α and 18S mRNA were measured by quantitative reverse transcription polymerase chain reaction analysis (Q-RTPCR) using the following primer pairs: forward primer (NRF1F) 5′–GTACAAGAGCATGATCCTGGA-3′ and reverse primer (NRF1R) 5′-GCTCTTCTGTGCGGACATC-3′, forward primer (TUFMF) 5′- ATTGGCACCGGTCTAGTCAC-3′ and reverse primer (TUFMR) 5′-TGTCCATCTAGCTGCCCTCT-3′, forward primer (TFAMF) 5′- AAGATTCCAAGAAGCTAAGGGTGA-3′ and reverse primer (TFAMR) 5′- CAGAGTCAGACAGATTTTTCCAGTTT-3′, forward primer (POLGF) 5′- GGAGGAGTTCCTGCTCACTG-3′ and reverse primer (POLGR) 5′- GAGGCAGCTTGAAAAACCAG-3′, forward primer (HIF1αF) 5′-CAAGAACCTACTGCTAATGC-3′ and reverse primer (HIF1αR) 5′-TTATGTATGTGGGTAGGAGATG-3′, forward primer (18SF) 5′- AGGAATTGACGGAAGGGCAC-3′ and reverse primer (18SR) 5′- GGACATCTAAGGGCATCACA - 3′. Equal amounts of cDNA for each sample were added to a prepared master mix (SYBR Green PCR Master mix; Applied Biosystems, Foster City, CA). Q-RTPCR reactions were performed on an ABI Prism 7900 sequence detection system (Applied Biosystems, Foster City, CA) as described previously [8]. The relative abundance of a transcript was represented by the threshold cycle of amplification (C_T_), which is inversely correlated to the amount of target RNA/first-strand cDNA being amplified. To normalize for equal amounts of the latter, we assayed the transcript levels of 18S gene. The comparative C_T_ method was calculated as per manufacturer’s instructions. The expression level of HIF1α relative to the baseline level was calculated as 2^–ΔC^_T_^(HIF1α)^, where ΔC_T_ is (average HIF1α C_T_ – average 18 s C_T_) and is C_T_ (average C_T_-untreated sample – average C_T_-treated sample).

### Statistical Analysis

All data are expressed as mean and standard deviation (SD) to indicate data variability. Experiments were repeated at least three times. Statistical analyses were performed by unpaired student’s t test. Differences were considered statistically significant at p<0.05.

## Results

### Tigecycline-resistant Cells Established by Sustained Drug Treatment

**Figure 1:**
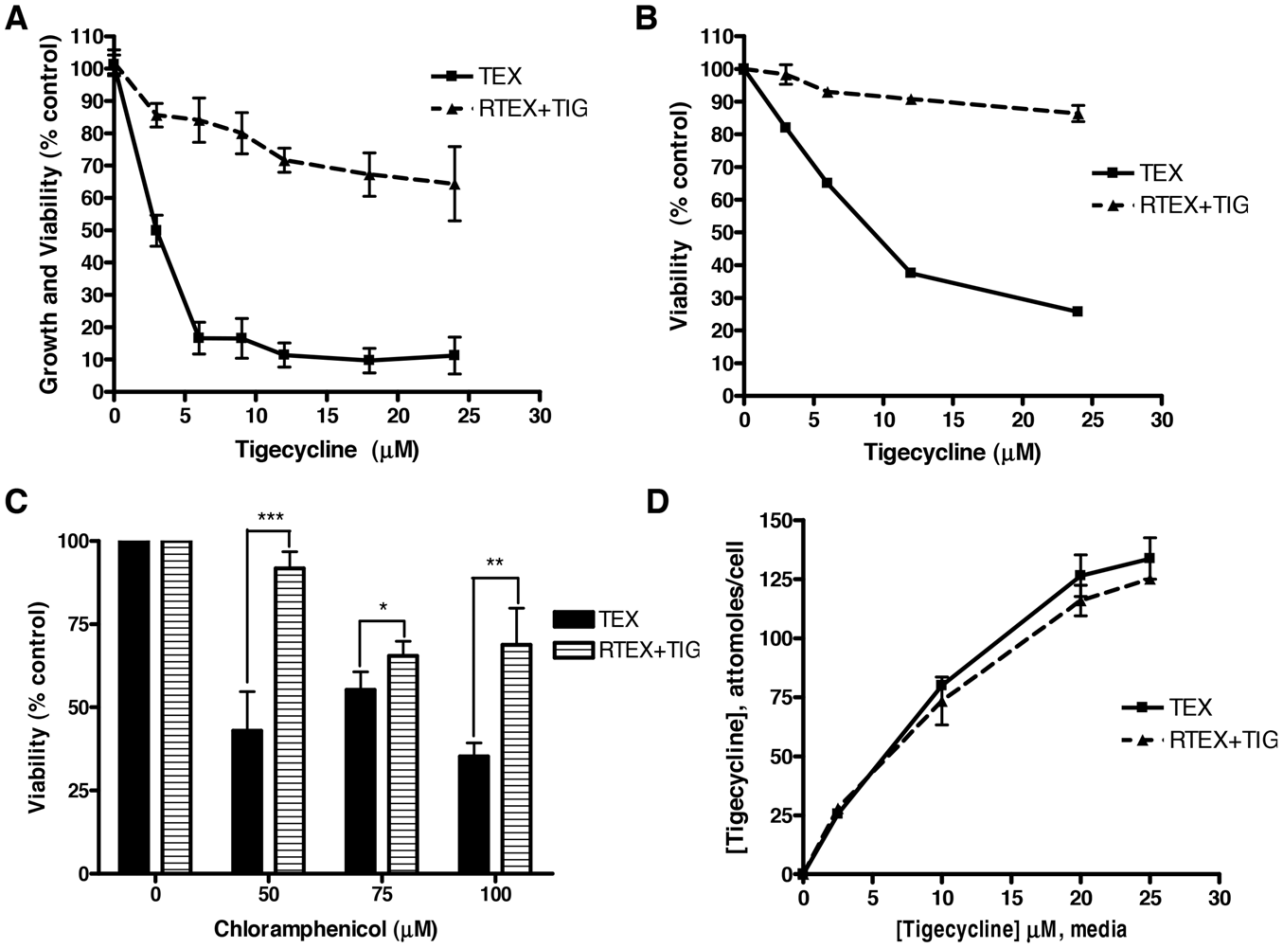
Tigecycline-resistant cells established by sustained drug treatment. TEX and RTEX+TIG cells were treated with increasing concentrations of tigecycline for 72 hours. **A** Cell growth and viability was measured by the sulforhodamine B assay. Data represent the mean ± SD percent from a representative experiment. **B** Cell viability was measured by Annexin V and PI staining and flow cytometry. Data represent the mean ± SD percent viable cells from a representative experiment. **C** TEX and RTEX+TIG cells were treated with increasing concentrations of chloramphenicol for 7 days. Cell viability was measured by trypan blue staining. Data represent the mean ± SD percent viable cells from three independent experiments. **D** Tigecycline was removed from the culture medium of RTEX+TIG cells for 3 hours. Then, RTEX+TIG and wild type TEX cells were treated with increasing concentrations of tigecycline for 6 hours. After treatment, cells were harvested and intracellular concentrations of tigecycline were determined by HPLC-UV. Data represent the mean ± SD intracellular concentration of tigecycline from a representative experiment.

To understand mechanisms of resistance to tigecycline in leukemia, we treated TEX leukemia cells with increasing concentrations of the drug in a step-wise manner over 4 months. Over this time, we selected a population of TEX cells capable of growth in the presence of 24 µM tigecycline (RTEX+TIG). RTEX+TIG were highly resistant to the drug with an IC_50_ greater than 24 µM (Figure 1A). In contrast, tigecycline reduced the growth and viability of wild type TEX cells with an IC_50_ value of 2.8±0.4 µM. We confirmed that tigecycline treatment induced cell death in the wild type, but not RTEX+TIG cells by Annexin V/PI staining and flow cytometry (Figure 1B). We also examined the effect of chloramphenicol, another known inhibitor of mitochondrial translation on the viability of TEX and RTEX + TIG cells (Figure 1C). Chloramphenicol induced cell death in TEX cells, but the RTEX+TIG cells were more resistant.

Alterations in intracellular drug levels through mechanisms such as decreased influx or increased export may explain tigecycline resistance in the RTEX+TIG cells. Therefore, we measured intracellular levels of tigecycline in wild type TEX and RTEX+TIG cells after drug treatment. For RTEX+TIG cells, tigecycline was removed from the medium for 3 hours, a time we demonstrated sufficient to eliminate tigecycline from the cells (data not shown). Wild type and resistant cells were then treated with increasing concentrations of tigecycline for 6 hours. After treatment, intracellular levels of tigecycline were measured by HPLC-UV. Intracellular levels of tigecycline were the same in the wild type TEX and RTEX+TIG cells (Figure 1D). Thus, the mechanism of RTEX+TIG resistance is not due to alterations in drug import or efflux.

### RTEX+TIG Cells have Defective Oxidative Phosphorylation

**Figure 2:**
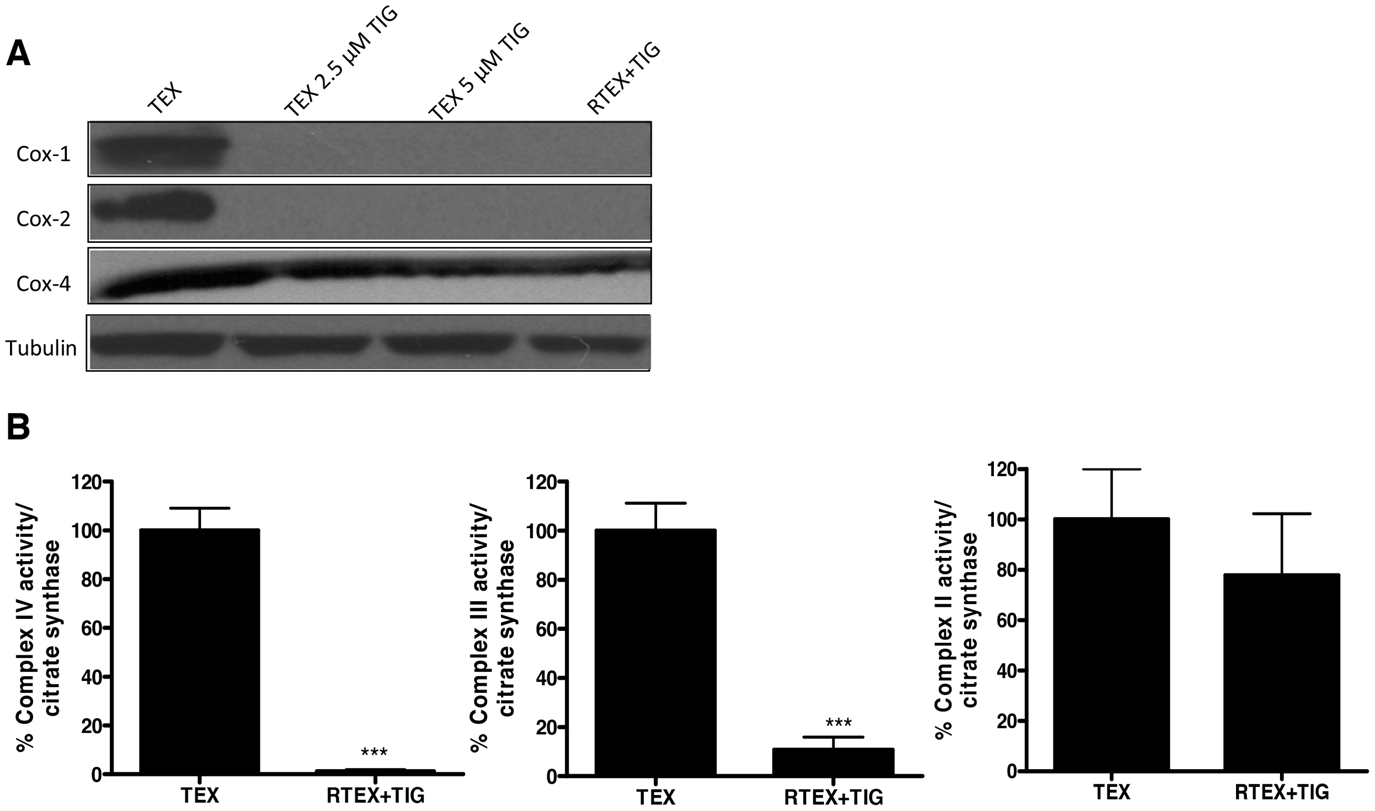
RTEX+TIG cells have undetectable levels and activity of mitochondrial respiratory chain complexes. Total proteins were extracted from untreated TEX and RTEX+TIG cells, as well as TEX cells treated with 2.5 µM and 5 µM tigecycline for 24 hours. **A** Levels of Cox-1, Cox-2, Cox-4 and tubulin were measured by immunoblotting. **B** Complex II, III and IV enzyme activity relative to citrate synthase activity was determined as described in the Materials and Methods section. Results shown as mean ± SD of three independent experiments.

Recently, we demonstrated that tigecycline inhibits mitochondrial protein translation in mammalian cells [4]. Therefore, we evaluated mitochondrial protein synthesis in the RTEX+TIG cells. Cytochrome C Oxidase-1 (Cox-1) and Cytochrome C Oxidase-2 (Cox-2) are translated by mitochondrial ribosomes [20] and are components of the complex IV respiratory chain enzyme, while Cytochrome C Oxidase-4 (Cox-4) comprises the same respiratory chain enzyme, but is translated by cytoplasmic ribosomes. Cox-1 and Cox-2 proteins were absent but Cox-4 expression was only slightly reduced in the RTEX+TIG cells (Figure 2A). We also evaluated the enzymatic activity of respiratory complexes III and IV, both of which contain proteins translated on mitochondrial ribosomes, and respiratory chain complex II, which does not contain mitochondrially-encoded subunits in its sub-structure [21]. In RTEX+TIG cells, complex III and IV activity was almost completely absent (p<0.001), but complex II activity was only slightly reduced (Figure 2B).

**Figure 3:**
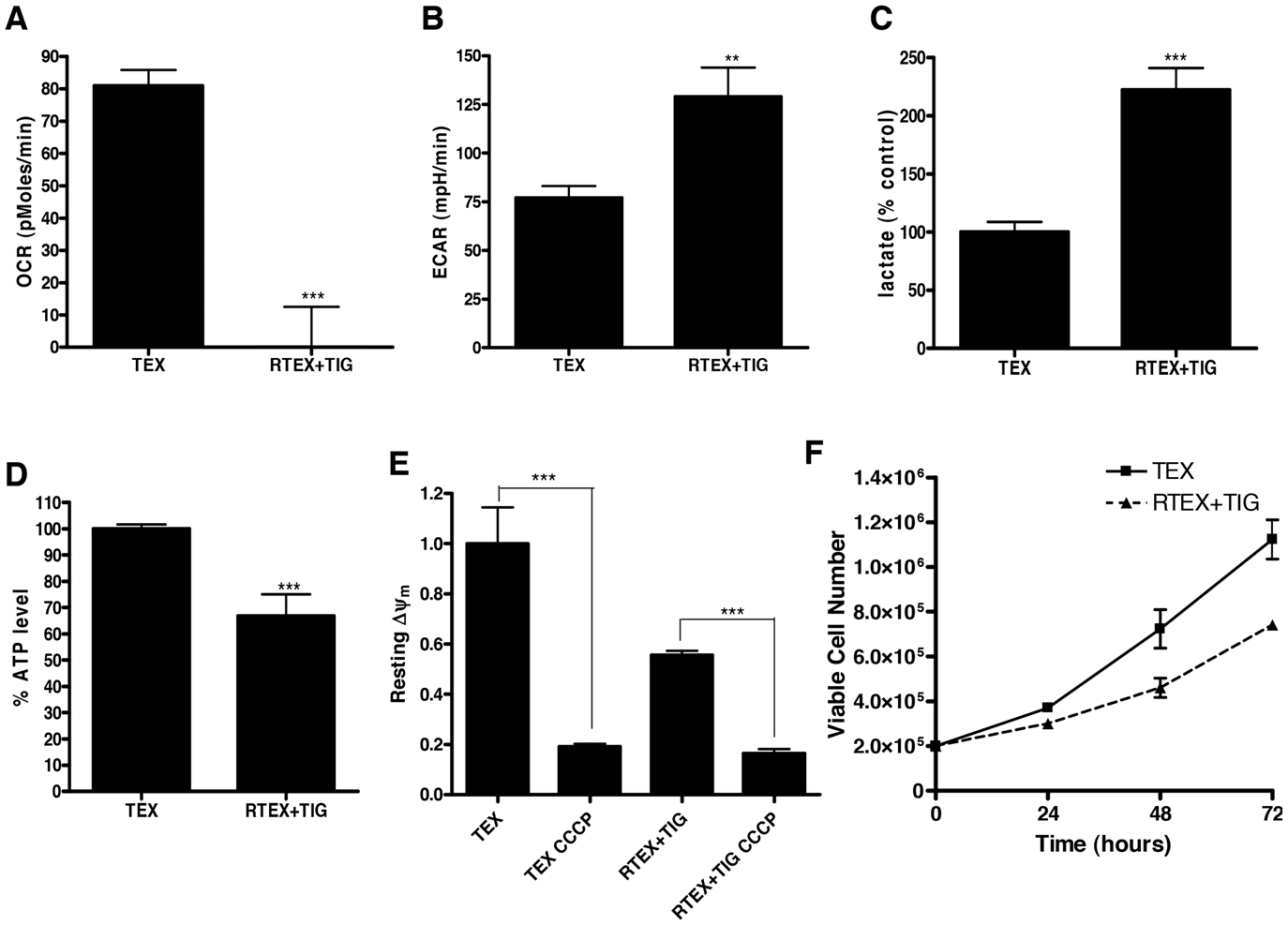
RTEX+TIG cells have defective oxidative phosphorylation. **A** Basal oxygen consumption rate of TEX and RTEX+TIG cells was measured with the Seahorse Metabolic Flux Analyzer as described in the Materials and Methods section. Results shown as mean ± SD of three independent experiments. **B** Basal extracellular acidification rate of TEX and RTEX+TIG cells was measured with the Seahorse Metabolic Flux Analyzer as described in the Materials and Methods section. Results shown as mean ± SD of three independent experiments. **C** Lactate production of TEX and RTEX+TIG cells (2×10^7^) was measured by NMR as described in the Materials and Methods section. Results shown as mean ± SD of independent experiments. **D** Intracellular ATP content of TEX and RTEX+TIG cells was measured as described in the Materials and Methods section. Results shown as mean ± SD from a representative experiment. **E** Resting mitochondrial membrane potential (Δψ, Red/Green ratio) was measured in TEX and RTEX+TIG cells before and after uncoupling the potential with CCCP. Cells were stained with JC-1 dye and analyzed by flow cytometry. Results shown as mean ± SD fluorescence intensity relative to TEX cells. **F** The number of viable TEX and RTEX+TIG cells was counted using trypan blue staining at 24, 48 and 72 hours. Results shown as mean ± SD viable cells from independent experiments.

Given the defects in the respiratory chain in RTEX+TIG cells, we examined their oxygen consumption rate (OCR), extracellular acidification rate (ECAR) as well as lactate production to determine the effects of chronic inhibition of mitochondrial protein synthesis on cellular metabolism. Oxygen consumption was undetectable (Figure 3A), while both ECAR and lactate production increased 2-fold (Figures 3B and 3C) in RTEX+TIG cells grown in the presence of tigecycline. We also measured levels of intracellular ATP in the paired cells and demonstrated that ATP levels were reduced by 33±3% in RTEX+TIG cells as compared to wild type TEX cells (Figure 3D). Of note, ECAR was not increased, but in fact reduced in wild type TEX cells treated for 12 hours with tigecycline (Figure S1).

The respiratory chain establishes a negative electrical gradient (ΔΨm) across the inner mitochondrial membrane. Therefore, we evaluated the ΔΨm in wild type and RTEX+TIG cells. Consistent with the reductions in oxidative phosphorylation, the resting mitochondrial membrane potential was significantly reduced in the RTEX+TIG cells (Figure 3E). However, treatment with the uncoupling agent CCCP reduced membrane potential even further, suggesting that RTEX+TIG cells maintained partial mitochondrial membrane potential despite oxidative phosphorylation dysfunction. We also determined the growth rates for both wild type TEX and resistant RTEX+TIG cell lines. RTEX+TIG cells had a slower rate of proliferation with a doubling time of 37.01±2.8 hours versus 26.79±3.07 hours for the wild type cells (Figure 3F).

**Figure 4:**
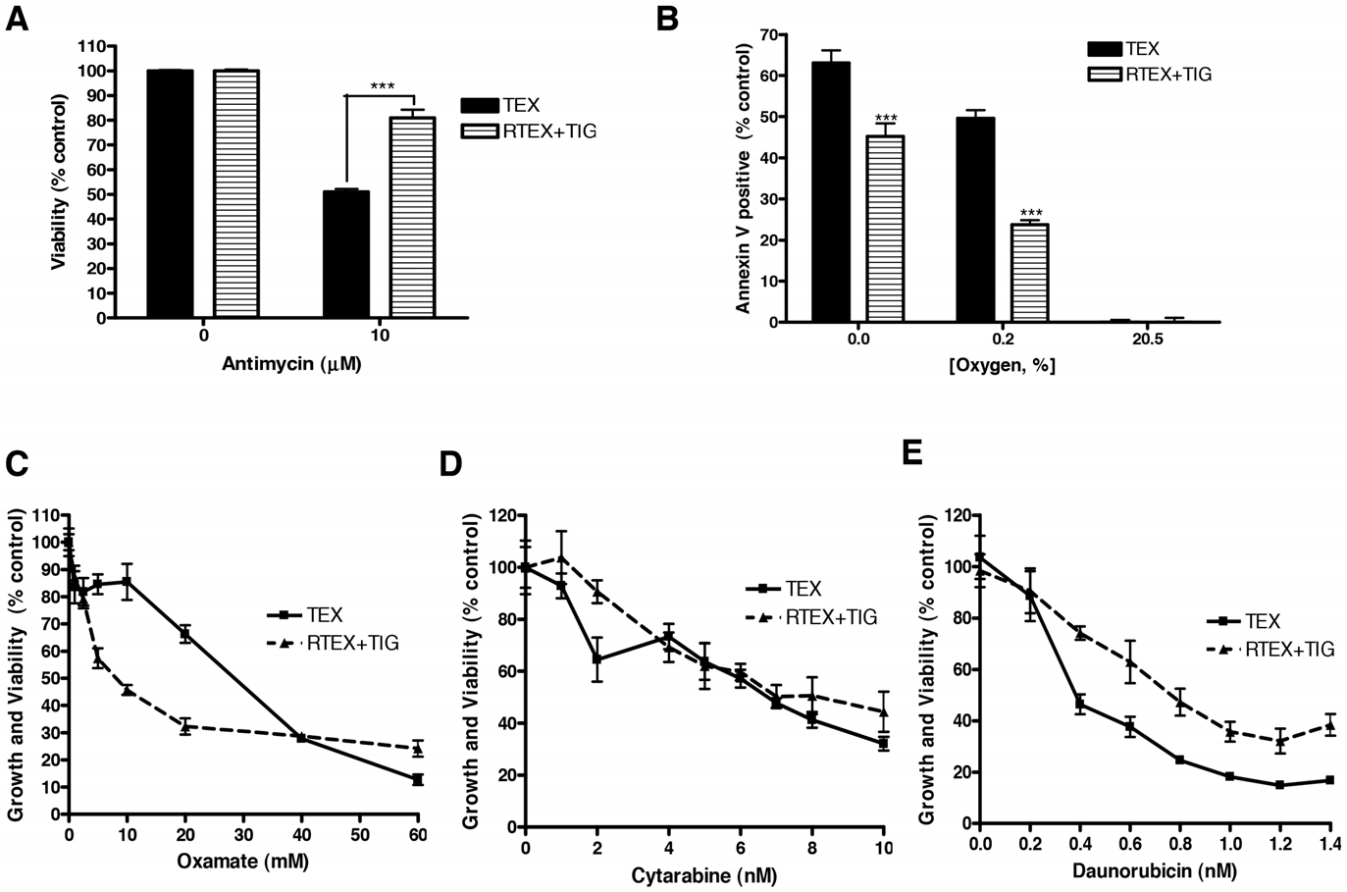
RTEX+TIG cells are not dependent on oxidative phosphorylation. **A** TEX and RTEX+TIG cells were treated with the complex III inhibitor, antimycin (10 µM) for 72 hours. After incubation, cell viability was measured by Annexin V and PI staining. Results are shown as the mean ± SD from a representative experiment. **B** TEX and RTEX+TIG cells were cultured in decreasing oxygen concentrations for 72 hours. After incubation, cell viability was measured by Annexin V and PI staining. Results are shown as the mean ± SD from independent experiments. **C** TEX and RTEX+TIG cells were treated with increasing concentrations of oxamate for 48 hours. After incubation, cell growth and viability were measured by the sulforhodamine B assay. Results are shown as the mean ± SD from a representative experiment. **D** TEX and RTEX+TIG cells were treated with increasing concentrations of cytarabine for 72 hours. After incubation, cell growth and viability were measured by the sulforhodamine B assay. Results are shown as the mean ± SD from a representative experiment. **E** TEX and RTEX+TIG cells were treated with increasing concentrations of daunorubicin for 72 hours. After incubation, cell growth and viability were measured by sulforhodamine B assay. Results are shown as the mean ± SD from a representative experiment.

Wild type TEX cells are dependent on oxidative phosphorylation and undergo cell death upon inhibition of mitochondrial protein translation with tigecycline. Therefore, we tested whether RTEX+TIG cells also had decreased functional dependence on oxidative phosphorylation. Compared to wild type TEX cells, RTEX+TIG cells were less sensitive to cell death after treatment with the complex III inhibitor antimycin (Figure 4A) or culture in anoxic or hypoxic conditions (Figure 4B). In contrast, RTEX+TIG cells were more sensitive to cell death after treatment with the glycolysis inhibitor oxamate (IC_50_ value of 9.4±0.9 mM), compared to wild type TEX cells (IC_50_ of 23.8±3.2 mM (Figure 4C). Thus, taken together, these results demonstrate that chronic inhibition of mitochondrial protein synthesis can select a population of cells no longer dependent on oxidative phosphorylation as they rely on glycolysis for their energy source.

We also compared the sensitivity of TEX and RTEX+TIG cells to standard chemotherapeutic agents used in the treatment of AML. Cytarabine was equally cytotoxic to TEX and RTEX+TIG cells (Figure 4D). In contrast, RTEX+TIG cells were more resistant to daunorubicin (Figure 4E). Thus, changes in cell metabolism affect sensitivity to some but not all chemotherapeutic agents.

### RTEX+TIG Cells have Altered Mitochondrial Mass and Structure

**Figure 5:**
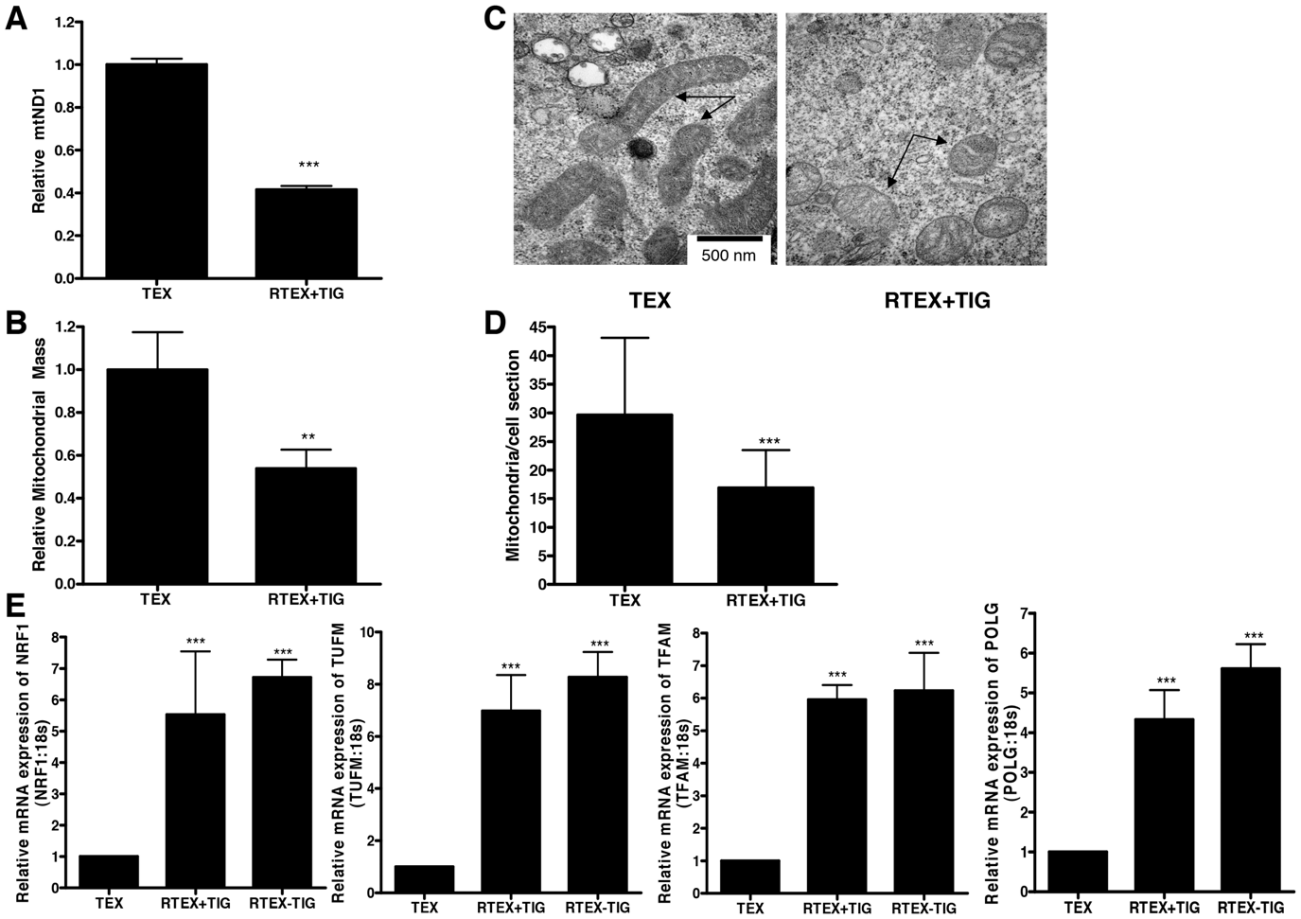
RTEX+TIG cells have altered mitochondrial mass and structure. **A** DNA was extracted from TEX and RTEX+TIG cells. Quantitative PCR was performed for mitochondrial *ND1* relative to the human globulin gene (*HGB*). Results shown as mean ± SD ratio of *ND1/HGB* compared to TEX cells from a representative experiment done in triplicate. **B** Mitochondrial mass was measured in TEX and RTEX+TIG cells by incubating cells with mitotracker Green FM dye and subsequent flow cytometry. Results shown as mean ± SD relative fluorescence intensity compared to TEX cells. A representative experiment is shown. **C** Mitochondrial morphology (arrows) was assessed in TEX and RTEX+TIG cells using transmission electron microscopy as described in the Materials and Methods section. Representative images taken at 50,000× are shown. The scale bar is 500 nm. **D** Mitochondria number was quantified by transmission electron microscopy. Data represent the mean number of the mitochondria reported as the cross-sectional area of individual mitochondria ± SD. Number of sections examined equals 16 in TEX and 20 in RTEX+TIG cells. **E** Total cellular RNA was isolated from TEX, RTEX+TIG, and RTEX-TIG cells. NRF1, TFAM, TUFM and POLG expression was measured relative to 18S RNA by real-time RT-PCR. Data represent the mean±SD HIF1α/18S expression relative to TEX cells from independent experiments.

Next, we examined changes in mitochondrial DNA, mass and structure in the RTEX+TIG cells. Although tigecycline is not known to directly affect mitochondrial DNA replication, mitochondrial DNA was decreased by 58±2% compared to wild type controls (Figure 5A). Likewise, mitochondrial mass, as measured by staining cells with Mitotracker Green FM, was reduced by 46±11% compared to wild type cells (Figure 5B). Note, unlike other conventional mitochondrial dyes Mitotracker Green FM stains mitochondria regardless of resting mitochondrial membrane potential [22]. We examined mitochondrial morphology by electron microscopy (Figure 5C). The mitochondria in RTEX + TIG cells were swollen with a translucent matrix and irregular cristae. In addition, we observed a decrease in the number of the mitochondria per cell section in RTEX+TIG cells (Figure 5D). Replication of mitochondrial DNA is regulated by genes encoded by the nuclear genome. Given the decreased mitochondrial mass in the RTEX+TIG cells, we measured the mRNA expression of nuclear genes involved in maintenance and replication of mtDNA as well as the regulation of mitochondrial mass (Figure 5E). Despite the reductions in the mitochondrial mass in RTEX+TIG cells, mRNA expression of POLG, TFAM, NRF1 and TUFM was significantly upregulated in RTEX+TIG cells compared to TEX cells.

### Transcriptome Profiling of RTEX+TIG Cells Identifies the Upregulation of a HIF1α-Controlled Gene Network

**Figure 6:**
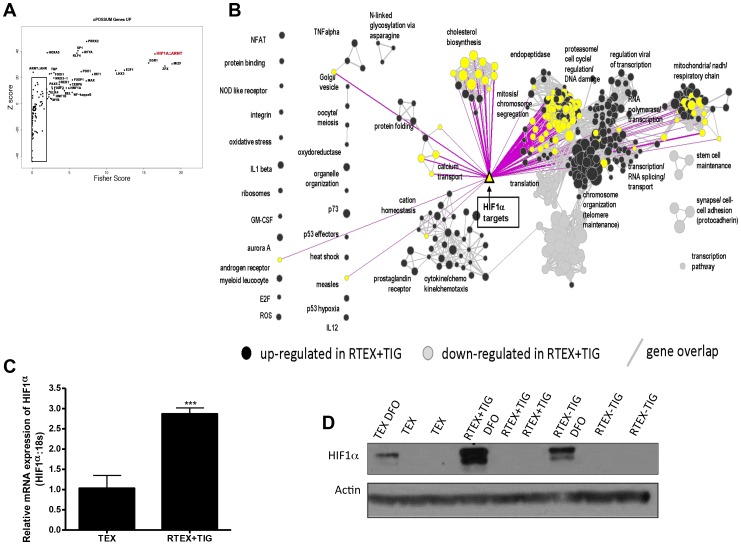
RTEX+TIG cells have increased expression of HIF1α. **A** Plot of the Fisher score (x-axis) vs. the z-score (y-axis) from the oPOSSUM transcription factor enrichment analysis of the list of genes over-expressed 1.5-fold or greater in RTEX+TIG cells compared to TEX cells. Each transcription factor is represented by a point on the graph; transcription factors whose binding sites are most strongly enriched within the promoters of the specified gene list have high scores (top right of plot). **B** Enrichment map visualization of the pathway enrichment analysis performed on the RNA sequencing dataset, which also maps the list of HIF1α transcriptional targets (yellow triangle), using a p-value cut-off of 0.001 and FDR cut-off of 0.1. Each circle (node) represents a gene set (pathway). Dark grey nodes are pathways enriched for genes up-regulated and light grey nodes are pathways enriched for genes down-regulated in RTEX+TIG cells, compared with wild type TEX cells. Yellow nodes represent gene sets that contain HIF1α transcriptional targets. Pathways (nodes) are connected when they overlap (i.e. they have genes in common), with line width corresponding to the number of shared genes (grey lines). Pink lines illustrate the connections between identified nodes and the HIF1α transcriptional target set. Node size is proportional to the GSEA normalized enrichment score (NES). **C** Total cellular RNA was isolated from TEX and RTEX+TIG cells. HIF1α expression was measured relative to 18S RNA by real-time RT-PCR. Data represent the mean±SD HIF1α/18S expression relative to TEX cells. **D** TEX, RTEX+TIG, and RTEX-TIG cells were treated with 100 µM desferoxamine (DFO) for 4 hours. After incubation, cells were harvested, total proteins were extracted, and levels of HIF1α and actin were measured by immunoblotting.

To understand the molecular basis by which RTEX+TIG cells were able to adapt to inhibition of mitochondrial protein synthesis, we conducted transcriptome profiling of RTEX+TIG cells and compared gene expression with parental TEX cells. From this analysis, we determined that approximately 2,400 genes were over-expressed in RTEX+TIG, while approximately 1,300 genes were under-expressed in RTEX+TIG cells (fold-difference cut-off 1.5). In an unbiased pathway enrichment analysis approach using GSEA, we determined that RTEX+TIG cells exhibited over-representation of genes involved in the electron transport chain and respiration as well as the hypoxic response. In addition, we detected upregulation of genes involved in cholesterol biosynthesis, proteasome activity, cytokine/cytokine receptor interaction, although the biological significance of these changes is unknown (Figure S2). Next, we examined the promoter sequences of genes that were upregulated 1.5-fold or greater in the RTEX+TIG cells and observed significant over-representation of binding sites for a variety of transcription factors involved in mitochondrial protein expression, cell growth and stress response (consistent with the results from our pathway analysis). Strikingly, genes with binding sites for both components of the HIF1α:HIF1β transcription factor complexes were among the most upregulated (predicted) and known direct HIF1α transcriptional targets [23] were also significantly enriched (Figures 6A–B).

### RTEX+TIG Cells have Increased Expression of HIF1α

Given the gene expression data, we examined levels of HIF1α expression in the RTEX+TIG and wild type cells and demonstrated that HIF1α mRNA was increased in RTEX+TIG cells compared to parental controls (Figure 6C). Of note, mutations in the coding region of HIF1α were not detected in our analysis of the transcriptome sequencing data. HIF1α protein was measured by immunoblotting after treating cells with the iron chelator desferoxamine that inhibits the iron-dependent enzyme prolyl hydroxylase and thereby prevents the degradation of HIF1α. RTEX+TIG cells also had increased expression of HIF1α protein compared to wild type TEX cells (Figure 6D).

### The Defective Oxidative Phosphorylation in RTEX+TIG is Reversible

**Figure 7:**
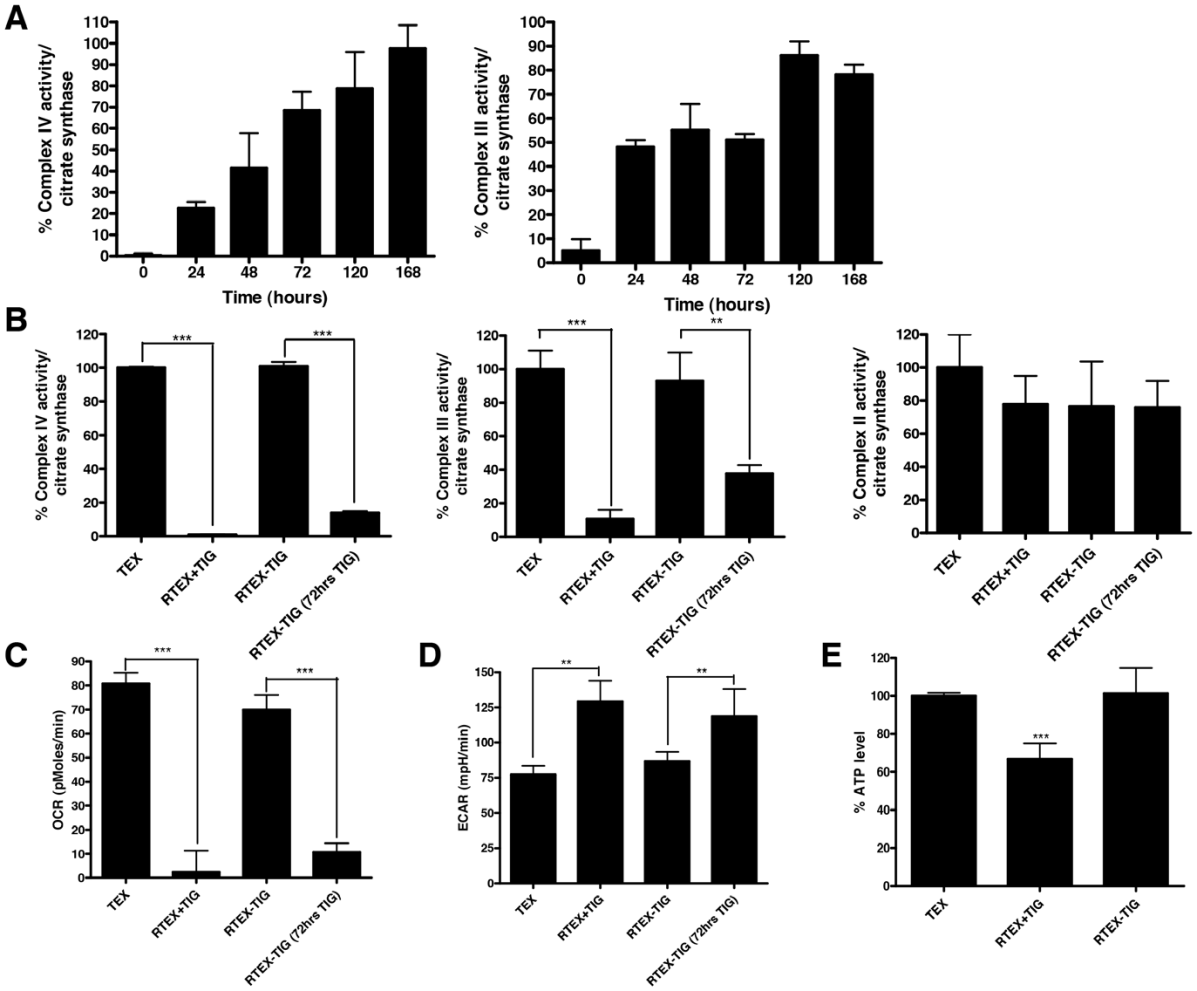
The defective oxidative phosphorylation in RTEX+TIG is reversible. Tigecycline was withdrawn from the culture media of RTEX+TIG cells for one week (RTEX-TIG). **A** Complex III and IV enzyme activity relative to citrate synthase activity was determined in RTEX+TIG cells upon the withdrawal of tigecycline (RTEX-TIG) at various time points as described in the Materials and Methods section. Results shown as mean ± SD of three independent experiments. **B** Cells were then re-treated with tigecycline (24 µM) for 72 hours (RTEX-TIG (72 hrs TIG)). Complex IV, III, and II activity was measured as described in the Materials and Methods section. Data represent the mean±SD from three independent experiments. **C** Cells were then re-treated with tigecycline (10 µM) for 72 hours (RTEX-TIG (72 hrs TIG)). Oxygen consumption rate was measured with the Seahorse Metabolic Flux Analyzer. Data represent the mean±SD from a representative experiment. **D** Cells were then re-treated with tigecycline (10 µM) for 72 hours (RTEX-TIG (72 hrs TIG)). Extracellular acidification rate was measured with the Seahorse Metabolic Flux Analyzer. Data represent the mean±SD from a representative experiments. **E** Intracellular ATP content was measured in TEX, RTEX+TIG and RTEX-TIG cells. Data represent the mean±SD relative to TEX cells from a representative experiments.

**Figure 8:**
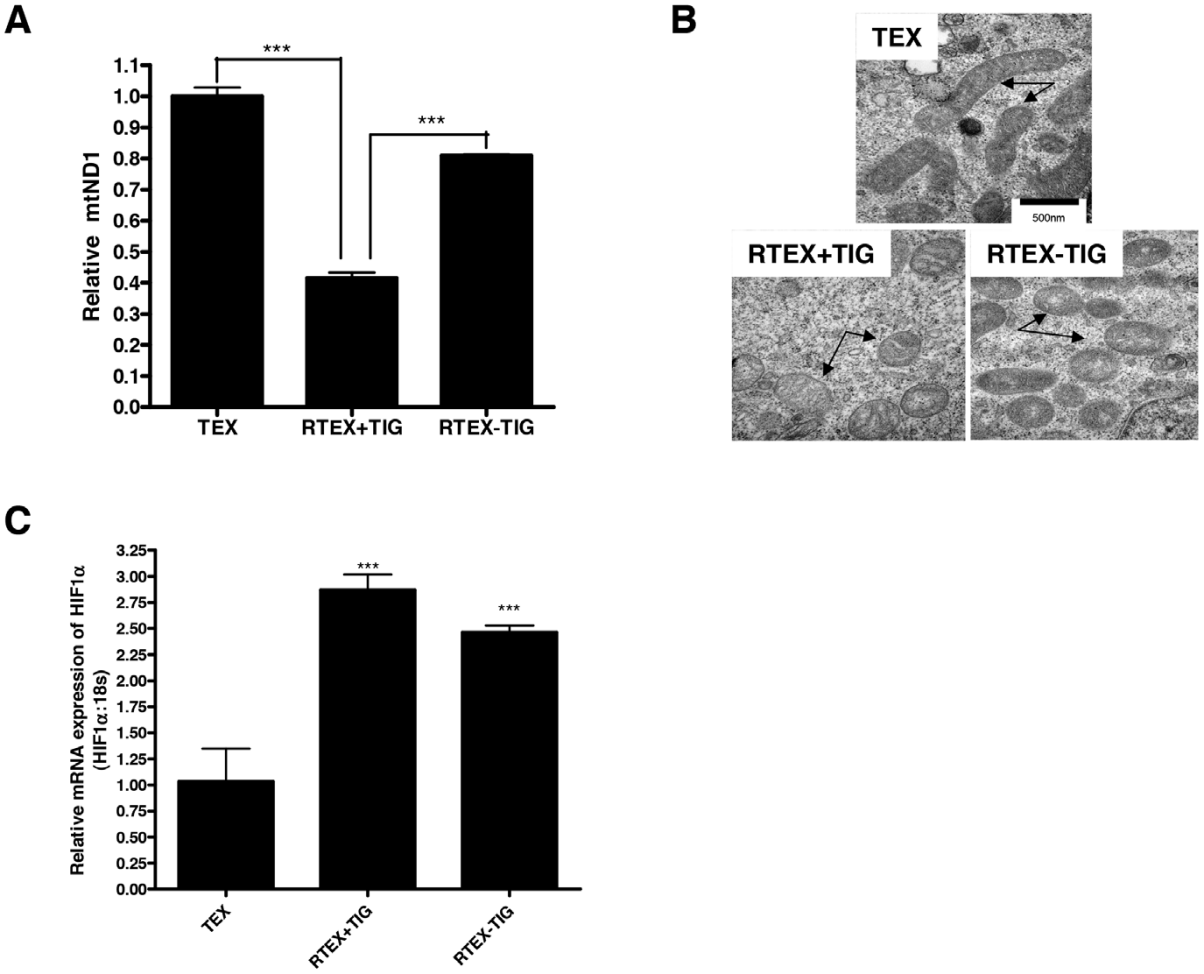
Changes in mitochondrial morphology and HIF1α expression in RTEX-TIG cells are irreversible. A DNA was extracted from TEX, RTEX+TIG cells, and RTEX-TIG cells. Quantitative PCR was performed for mitochondrial *ND1* relative to the human globulin gene (*HGB*). Results shown as mean ± SD ratio of *ND1/HGB* compared to TEX cells from a representative experiment done in triplicate. **B** Mitochondrial morphology (arrows) was assessed in TEX, RTEX+TIG and RTEX-TIG cells using transmission electron microscopy as described in the Materials and Methods section. Representative images taken at 50,000× are shown. The scale bar is 500 nm. **C** Total cellular RNA was isolated and HIF1α expression was measured relative to 18S RNA by real-time RT-PCR. Data represent the mean±SD HIF1α/18S expression relative to TEX cells.

In classic rho-zero cells, elimination of the mitochondrial DNA leads to a permanent defect in oxidative phosphorylation and an irreversible change in metabolism. Tigecycline is a reversible inhibitor of ribosomal function, so we assessed the reversibility of the metabolic abnormalities in RTEX+TIG cells by withdrawing tigecycline from the cell culture medium for one week. Withdrawal of tigecycline for one week reversed the metabolic defects seen in RTEX+TIG cells. We measured the restoration of Complex III and IV enzymatic over time and demonstrated that complex activity gradually restored to wild type levels within 7 days (Figures 7A and B). In addition, OCR (Figure 7C), ECAR (Figure 7D), ATP levels (Figure 7E), and mitochondrial DNA copy number (Figure 8A) also reverted to wild type levels. Despite reversal of the metabolic phenotype, the structural defects in the mitochondria persisted. One week after the withdrawal of tigecycline, the mitochondria remained swollen and translucent in RTEX-TIG cells (Figure 8B). In addition, levels of NRF1, TUFM, TFAM, and POLG also remained increased (Figure 5E). HIF1α mRNA and protein also remained elevated compared to wild type control (Figures 8C and 6D). Yet, upon re-treatment with tigecycline for just 72 hours, oxidative phosphorylation could again be abolished and glycolysis increased (Figures 7B–D). Of note, upon re-treatment with tigecycline, RTEX cells remained resistant to the drug (Figure S3).

## Discussion

The glycylcycline tigecycline exerts its anti-bacterial effects by inhibiting protein translation in bacteria. Specifically, it binds reversibly to the A-site of the 30S subunit of the bacterial ribosome, and thereby prevents docking of aminoacyl-tRNAs [24]. Recently, we demonstrated that tigecycline is selectively cytotoxic to AML cells and AML stem cells and this cytotoxicity is attributable to inhibition of mitochondrial protein translation [4]. Here, we evaluated mechanisms of resistance to tigecycline in leukemia cells by selecting a population of TEX cells resistant to the drug. We demonstrated that the cells resistant to tigecycline were also cross-resistant to chloramphenicol, another inhibitor of mitochondrial translation. The cross-resistance to chloramphenicol further supports a functional effect of tigecycline on the mitochondrial ribosome and mitochondrial protein translation. Mitochondrial protein translation was inhibited in the resistant cells and oxidative phosphorylation was undetectable. The resistant cells adapted to inhibition of oxidative phosphorylation by upregulating glycolysis. Thus, this mechanism of resistance supports our previous study demonstrating that tigecycline’s anti-leukemic activity was related to its effects on mitochondrial protein synthesis.

Compared to the parental cells, the RTEX+TIG cells were resistant to daunorubicin, yet were equally sensitive to cytarabine. A study by Lo *et al*., demonstrated that daunorubicin can target mitochondria, as rho-zero cells devoid of mtDNA were more resistant to the effects of daunorubicin compared to the wild type cell line [25]. While additional studies are needed to determine the mechanism of cross-resistance to daunorubicin and not cytarabine, it is interesting to speculate that the activity of some not all chemotherapeutic agents is dependent on a cell’s metabolic state.

The mitochondrial protein translation machinery is distinct compared to protein synthesis in the cytoplasm. For example, mitochondria have only 22 tRNAs to read all the mRNA codons, while the wobble hypothesis predicts that a minimum of 31 tRNAs are necessary to interpret the 61 mRNA codons to make 20 amino acids [1], [26]. In addition, only two initiation factors, mtIF2 and mtIF3, and three elongation factors, mtEF-Tu, mtEF-Ts and mtEFG, are found in mitochondria [27], [28]. In contrast, protein synthesis in the cytoplasm is much more complex, with at least nine initiation factors (eIF1–eIF6) and two elongation factors, eEF-1 and eEF-2 [29], [30]. Finally, mitochondria also contain unique release factors, mtRF1 and mtRF1a, that terminate translation by recognizing the stop codon, and a unique recycling factor, mtRRF that enables dissociation of mitochondrial ribosomal subunits, tRNA and mRNA molecules [27], [28]. Thus, targeting some of these unique components of mitochondrial protein synthesis may also have preferential cytotoxicity towards leukemia cells and may be novel therapeutic strategies for this disease. Our current study, however, highlights a potential mechanism of resistance which may be of concern as AML patients may be treated with these new therapies. Over time, populations of resistant cells may be selected that have adapted to the inhibition of mitochondrial translation and oxidative phosphorylation by increasing glycolysis. Interestingly, while tigecycline treatment inhibited the expression of proteins encoded by mitochondrial DNA and translated in the mitochondria, our gene expression analysis demonstrated upregulation of genes encoded by nuclear DNA that contribute to the mitochondrial respiratory chain and mitochondrial function. Potentially, upregulation of these genes represents a compensatory response to sustained inhibition of mitochondrial translation.

RTEX+TIG cells had almost complete loss of complex III and IV activity. Yet, they were able maintain partial mitochondrial membrane potential. A similar phenomenon has been observed in the classical rho-zero cells that utilize glycolytic ATP and incompletely assembled complex V to reverse ATP transport into mitochondria and hydrolyze glycolytic ATP to translocate protons into the intermembrane space, thus establishing an electrochemical gradient [31]. However, further studies in our cell lines will be required to understand how partial mitochondrial membrane potential is maintained despite inhibiting mitochondrial translation.

Although tigecycline is not known to directly affect mitochondrial DNA replication, the abundance of mitochondrial DNA and mitochondrial mass was reversibly decreased in the RTEX+TIG cells compared to wild type controls. Despite the reduction in mtDNA our data demonstrated a significant increase in the expression of the nuclear genes that regulate mtDNA replication and transcription. This is most likely a compensatory mechanism used by a cell to increase mtDNA replication due to the reduction in mitochondrial biogenesis that was observed as a reduction in the number of mitochondria. Previous studies have demonstrated that HIF1α negatively regulates mitochondrial DNA copy number and mitochondrial biogenesis [32]. However, upon withdrawal of tigecycline, DNA copy number reverted to levels near wild type, but HIF1α remained elevated. Thus, additional mechanisms related to disrupting mitochondrial protein translation and oxidative phosphorylation can feedback on mitochondrial DNA replication.

The removal of tigecycline from RTEX+TIG cells restored the metabolic phenotype to that of the wild type. However, mitochondria remained swollen and translucent upon the withdrawal of the drug, suggesting that despite the similar metabolic phenotype RTEX-TIG cell line is distinctly different from the wild type TEX cells. We suggest that these resistant cells have acquired the means to adapt to different metabolic environments. While unlikely, we cannot fully exclude the possibility that a small population of wild type TEX cells remains within the population of RTEX+TIG resistant cells. Upon withdrawal of tigecycline, these wild type cells might be able to grow more rapidly and become the dominant population over 7 days.

Molecularly, RTEX+TIG cells have increased levels of HIF1α mRNA and protein compared to wild type cells. HIF1α is a key modulator of the transcriptional responses to hypoxic stress, as HIF1α:HIF1β heterodimers bind hypoxia response elements (HREs) in the genome to initiate transcription of genes that encode for glycolytic enzymes and repress oxidative phosphorylation [33]. Regulation of HIF1α at the protein level is well studied. For example, under normoxic conditions HIF1α is hydroxylated at a conserved prolyl residue by prolyl hydroxylase. Hydroxylated HIF1α is then recognized and ubiquitinated by the von Hippel-Lindau (VHL) E3 ubiquitin ligase complex and targeted for degradation by the proteasome. Under hypoxic conditions or in the presence of increased reactive oxygen species or iron chelators, prolyl hydroxylase activity is inhibited, and HIF1α is then not hydroxylated, ubiquitinated or degraded, thus HIF1α protein is stabilized [34]. HIF1α stabilization can also be achieved independent of O_2_ concentration.

Although upregulation of HIF1α mRNA and protein were observed in the RTEX+TIG cells, further studies will be necessary to determine the functional importance of HIF1α upregulation in explaining the observed metabolic phenotype in the RTEX+TIG cells. Interestingly, upon withdrawal of tigecycline, HIF1α remained elevated, but the cells reverted to greater utilization of oxidative phosphorylation. Potentially, the ability to switch between glycolytic and oxidative phosphorylation states is regulated by the rate of degradation of this protein. In our studies, we assessed levels of HIF1α after blocking its degradation. Further studies would be necessary to understand rates of HIF1α degradation in these paired lines. Further studies will also be required to understand the molecular mechanisms that allow TEX cells to become resistant to tigecycline and upregulate glycolysis.

While the regulation of HIF1α protein degradation has been studied extensively, the regulation of HIF1α mRNA is less well understood. Reported regulators of HIF1α mRNA expression include NF-κB, homeodomain-interacting protein kinase-2 (HIPK2), and heat shock factor proteins 2 and 4 [35], [36], [37]. Further studies will be necessary to understand the mechanism by which HIF1α mRNA is upregulated in the tigecycline resistant cells. However, it is interesting to speculate that the upregulation of HIF1α mRNA may be related to increased levels of heat shock factor proteins 2 and 4, as these proteins modulate cellular response to stress.

In summary, we have generated reversible leukemic cell line through sustained inhibition of mitochondrial protein synthesis. These data provide further insight into how cells cope with metabolic stress.

## Supporting Information

Figure S1 **The effect of tigecycline treatment on the basal extracellular acidification rate (ECAR) of TEX cells.** TEX cells were treated with increasing concentrations of tigecycline for 12 hours. Basal extracellular acidification rate of TEX cells was measured with the Seahorse Metabolic Flux Analyzer as described in the Materials and Methods section. Results shown as mean ± SD.
(TIF)

Figure S2 **Pathway and network analysis in TEX and RTEX+TIG cells using gene set enrichment analysis.** Enrichment map visualization of the gene set enrichment analysis performed on the RNA sequencing dataset, using a p-value cut-off of 0.001 and FDR cut-off of 0.1. Each circle (node) represents a gene set (pathway). Dark grey nodes are pathways enriched for genes up-regulated and light grey nodes are pathways enriched for genes down-regulated in RTEX+TIG cells, compared with wild type TEX cells. Pathways (nodes) are connected when they overlap (i.e. they have genes in common), with line width corresponding to the number of shared genes (grey lines). Node size is proportional to the GSEA normalized enrichment score (NES).
(TIF)

Figure S3 **RTEX-TIG cells retain resistance to tigecycline.** TEX and RTEX-TIG cells were treated with increasing concentrations of tigecycline for 72 hours. Cell viability was measured by Annexin V and PI staining and flow cytometry. Data represent the mean ± SD percent viable cells from a representative experiment.
(TIF)
