# Supplementary figures and images for: Metabolic Adaptation to Chronic Inhibition of Mitochondrial Protein Synthesis in Acute Myeloid Leukemia Cells
Source: PLoS One. 2013 Mar 8;8(3):e58367. doi: 10.1371/journal.pone.0058367 (PMC3592803; doi:10.1371/journal.pone.0058367)

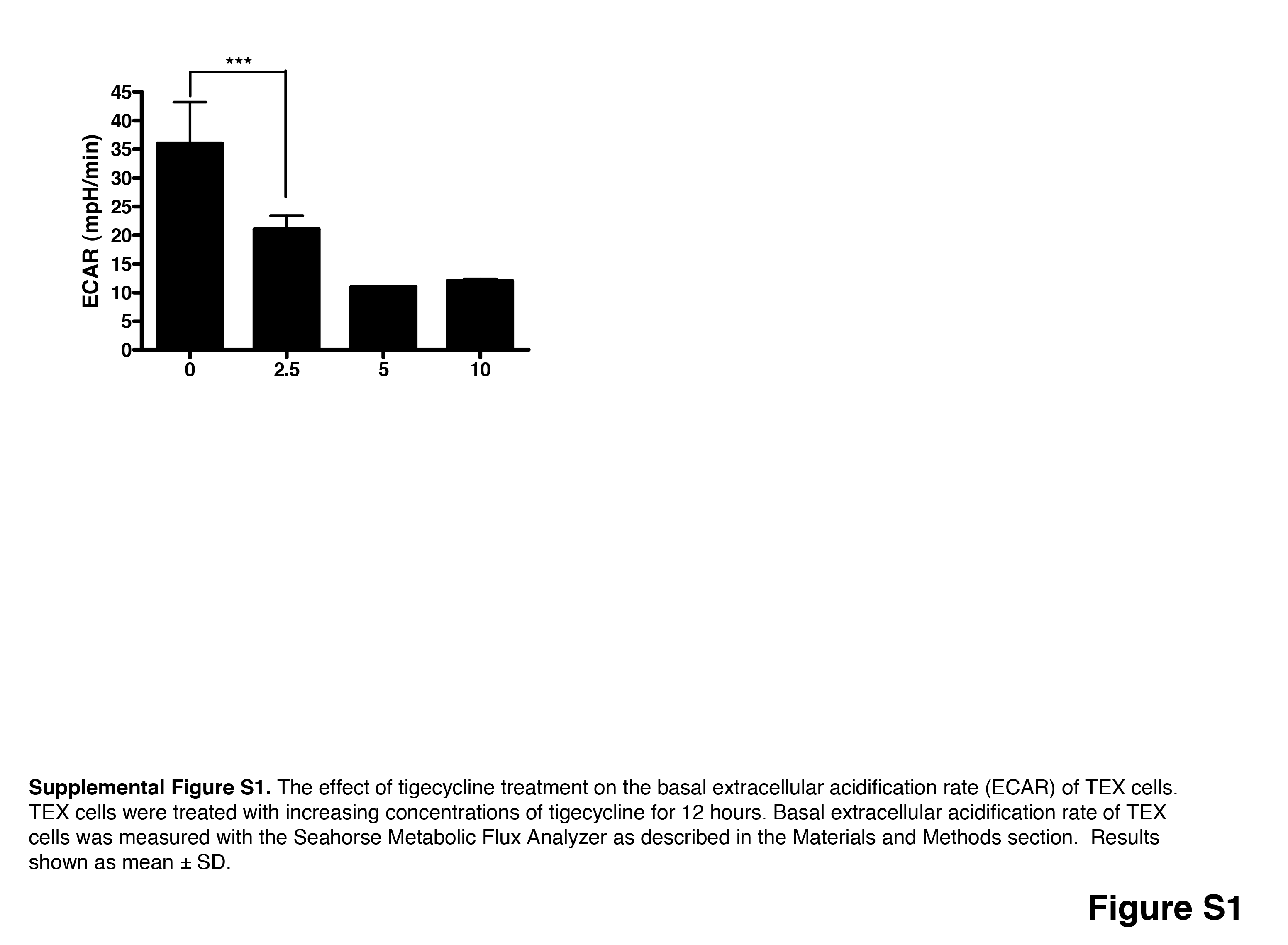

Supplement: Figure S1 — The effect of tigecycline treatment on the basal extracellular acidification rate (ECAR) of TEX cells. TEX cells were treated with increasing concentrations of tigecycline for 12 hours. Basal extracellular acidification rate of TEX cells was measured with the Seahorse Metabolic Flux Analyzer as described in the Materials and Methods section. Results shown as mean ± SD. (TIF) [file pone.0058367.s001.tif]

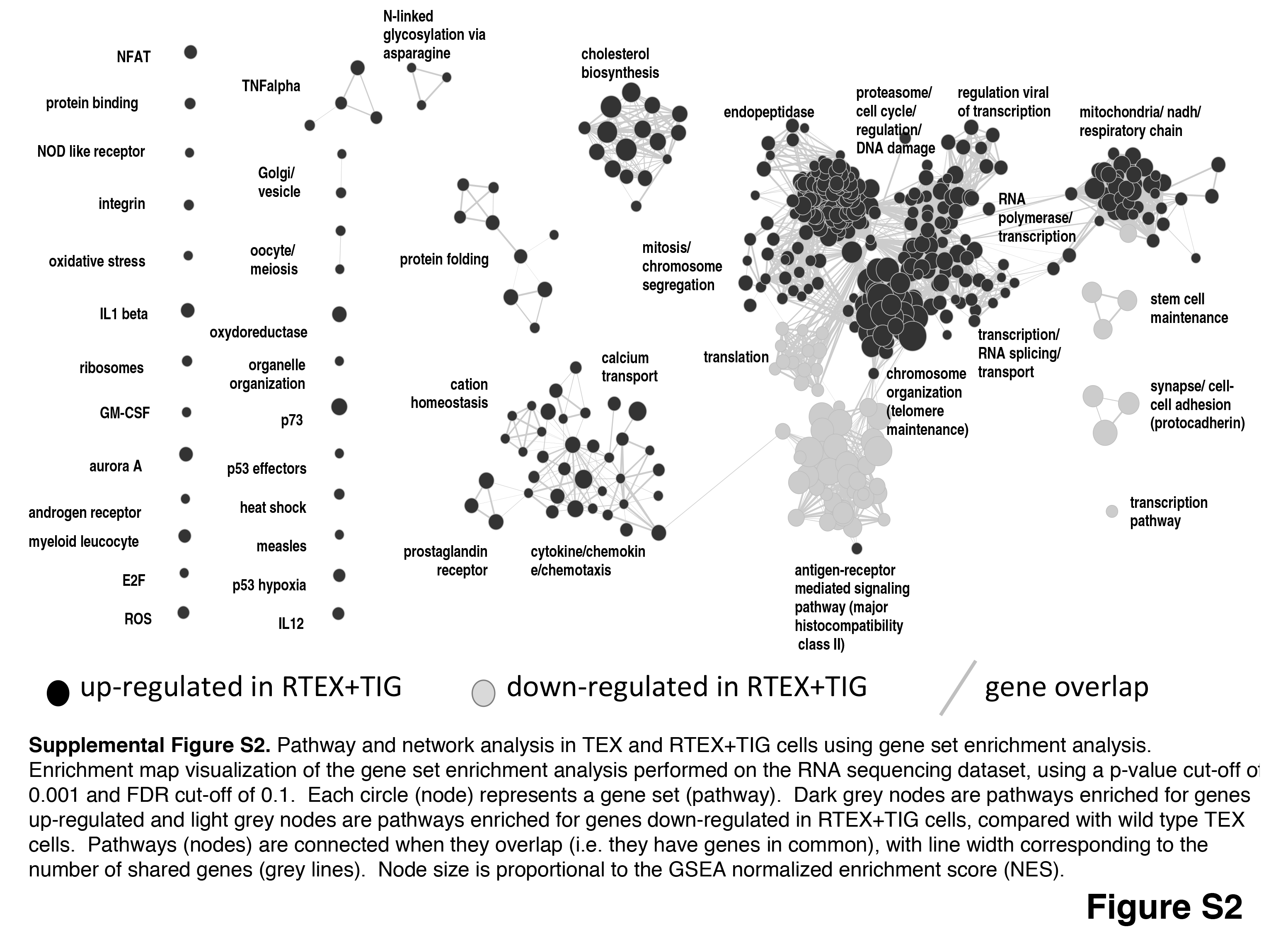

Supplement: Figure S2 — Pathway and network analysis in TEX and RTEX+TIG cells using gene set enrichment analysis. Enrichment map visualization of the gene set enrichment analysis performed on the RNA sequencing dataset, using a p-value cut-off of 0.001 and FDR cut-off of 0.1. Each circle (node) represents a gene set (pathway). Dark grey nodes are pathways enriched for genes up-regulated and light grey nodes are pathways enriched for genes down-regulated in RTEX+TIG cells, compared with wild type TEX cells. Pathways (nodes) are connected when they overlap (i.e. they have genes in common), with line width corresponding to the number of shared genes (grey lines). Node size is proportional to the GSEA normalized enrichment score (NES). (TIF) [file pone.0058367.s002.tif]

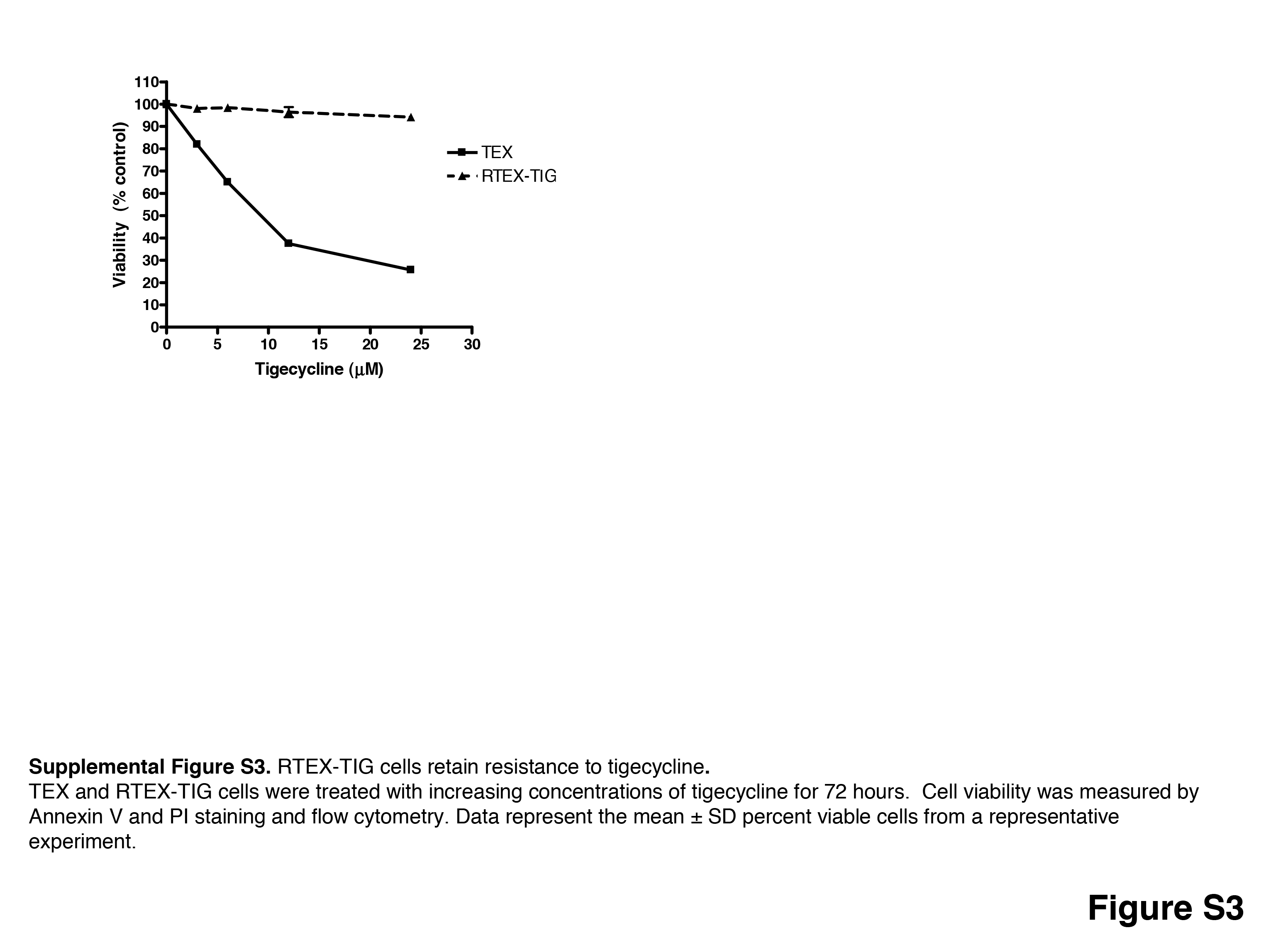

Supplement: Figure S3 — RTEX-TIG cells retain resistance to tigecycline. TEX and RTEX-TIG cells were treated with increasing concentrations of tigecycline for 72 hours. Cell viability was measured by Annexin V and PI staining and flow cytometry. Data represent the mean ± SD percent viable cells from a representative experiment. (TIF) [file pone.0058367.s003.tif]
